# T Cell Factor-1 Controls the Lifetime of CD4+ CD8+ Thymocytes *In Vivo* and Distal T Cell Receptor α-Chain Rearrangement Required for NKT Cell Development

**DOI:** 10.1371/journal.pone.0115803

**Published:** 2014-12-23

**Authors:** Archna Sharma, Rosa Berga-Bolaños, Jyoti Misra Sen

**Affiliations:** Immune Cells and Inflammation Section, National Institute on Aging, National Institutes of Health, Baltimore, MD, 21224, United States of America; Oklahoma Medical Research Foundation, United States of America

## Abstract

Natural killer T (NKT) cells are a component of innate and adaptive immune systems implicated in immune, autoimmune responses and in the control of obesity and cancer. NKT cells develop from common CD4+ CD8+ double positive (DP) thymocyte precursors after the rearrangement and expression of T cell receptor (TCR) Vα14-Jα18 gene. Temporal regulation and late appearance of Vα14-Jα18 rearrangement in immature DP thymocytes has been demonstrated. However, the precise control of lifetime of DP thymocytes *in vivo* that enables distal rearrangements remains incompletely defined. Here we demonstrate that T cell factor (TCF)-1, encoded by the *Tcf7* gene, is critical for the extended lifetime of DP thymocytes. TCF-1-deficient DP thymocytes fail to undergo TCR Vα14-Jα18 rearrangement and produce significantly fewer NKT cells. Ectopic expression of Bcl-x_L_ permits Vα14-Jα18 rearrangement and rescues NKT cell development. We report that TCF-1 regulates expression of RORγt, which regulates DP thymocyte survival by controlling expression of Bcl-x_L_. We posit that TCF-1 along with its cofactors controls the lifetime of DP thymocytes *in vivo*.

## Introduction

T cell development in the thymus is characterized by the rearrangement and expression of T cell receptor (TCR) α- and β-chains. TCRβ chain rearranges first in CD4^−^ CD8^−^ double negative (DN) cells and is expressed on the cell surface with a pre-Tα chain and the components of CD3 complex. Thymocytes that express a pre-Tα/TCRβ chain complex transition to the immature CD4^+^ CD8^+^ double positive (DP) stage and initiate the rearrangement of the TCRα chains. TCRα chain rearrangement persists until a productive interaction between the TCRαβ complex and the MHC complex is registered by the positive selection of the T cell. If the cell fails to be positively selected, a secondary Vα-Jα rearrangement proceeds to replace the failed TCRα chain [1]. This temporal process is initiated at the 5′ end of the Jα cluster and progresses through the 3′ Jαs during the lifetime of the DP thymocyte [2]. Natural killer T (NKT) cells develop from DP thymocytes with Vα14-Jα18 TCRα chain rearrangement paired with Vβ8, Vβ7 or Vβ2. The resulting limited TCR repertoire recognizes glycolipid antigens presented by the MHC class I-like molecule CD1d [3]. NKT cells produce cytokines when TCR is stimulated with their ligand α-galactosylceramide (α-GalCer) [4]–[6]. As Vα14-Jα18 rearrangement is a temporally distal event, deletion of genes such as transcription factor RORγt, that limit the lifetime of DP thymocytes, generally lead to impaired NKT cell development and immunity [2], [3]. However, the transcriptional program that controls the lifetime of DP thymocytes *in vivo* remains to be fully defined.

TCF-1, encoded by the *Tcf7* gene, and co-factor β-catenin are evolutionarily conserved transcription factors that work together and separately with other factors to regulate gene expression. In T cells, TCF-1 is induced by the Notch signaling pathway and participates in T cell commitment in the thymus [7], [8]. β-Catenin is ubiquitously expressed and in T cells is augmented in response to TCR signals [9]. Cooperating together and functioning independently, these transcription factors regulate gene expression that control critical aspects of conventional T cell development and function [10]–[13]. In addition, we have demonstrated that TCF-1 and β-catenin regulate the generation of innate-like CD8 (iCD8) thymocytes [14]. Transcription factor RORγt was shown to be a target of TCF-1 and shown to regulate thymocyte survival by controlling expression of Bcl-x_L_ [15]. TCF-1 and β-catenin also regulate thymocyte survival *in vitro* [15], [16]. However, their role in controlling the lifetime of DP thymocyte *in vivo* has not been defined. In particular, it remains to be demonstrated if TCF-1 and β-catenin regulate distal TCRα chain rearrangements and control NKT cell development.

In this study, we demonstrate that TCF-1 deletion results in significantly decreased NKT cells in the thymus. Enforced expression of Vα14-Jα18 TCR (Vα14) transgene resulted in the rescue of NKT cells, indicating that the reduction in the frequency of NKT cells was in part due to a failure to rearrange the Vα14-Jα18 TCRα chain. Ectopic expression of Bcl-x_L_ also rescued the frequency of Vα14-Jα18 rearrangement and the NKT cell subset. Finally, we show that *in vivo* TCF-1 controls DP thymocyte lifetime by prompting expression of RORγt as TCF-1-deficient DP thymocytes failed to express RORγt. These studies demonstrate that the decrease in the frequency and number of NKT cells was due to a decrease in the lifetime of DP thymocytes in TCF-1-deficent mice. We posit that TCF-1 controls the lifetime of DP thymocytes *in vivo*.

## Results

### TCF-1 is expressed in NKT cells in a developmentally relevant manner

**Figure 1:**
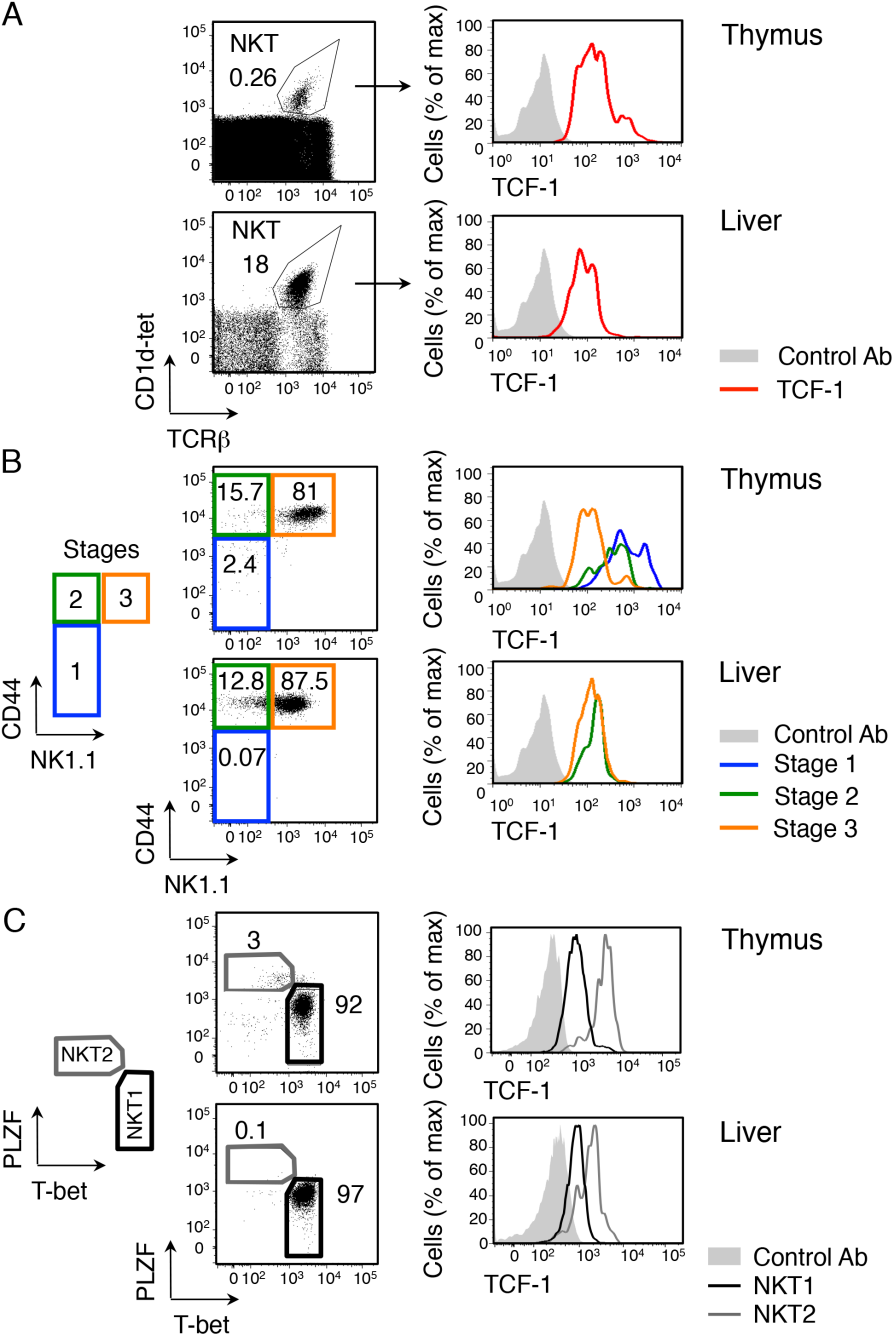
TCF-1 is expressed in NKT cells in a developmentally relevant manner. (**A**) Flow cytometry of thymocytes (**top**) and hepatic lymphocytes (**bottom**) from wild-type (WT) C57BL/6 mice showing TCF-1 intracellular expression versus the control antibody (**right**) in NKT cells (gated in **left** as CD1d-tetramer+ TCRβ+ cells, numbers indicate percentage of NKT cells). (**B**) Flow cytometry of CD1d-tetramer positive thymocytes (**top**) and hepatic lymphocytes (**bottom**) from WT mice stained with anti-CD44 and anti-NK1.1 to assess NKT developmental stages 1–3, as depicted in **left**. **Right**, TCF-1 intracellular expression among stages. Data are from a representative experiment out of four WT mice analyzed. (**C**) Flow cytometry of CD1d-tetramer positive thymocytes (**top**) and hepatic lymphocytes (**bottom**) from WT mice showing TCF1 intracellular expression versus the control antibody (**right**) in NKT1 and NKT2 NKT cells (gated in **left**, numbers indicate percentage of NKT cells). Data are from a representative experiment out of four WT mice analyzed.

To determine the role of TCF-1 in NKT cell generation, we first analyzed the expression of this protein by flow cytometry in NKT cells from control wild-type (WT) mice after staining target cells from lymphoid organs with anti-TCF-1, anti-TCRβ and CD1d tetramers loaded with glycolipid α-GalCer analogue PBS-57 (CD1d-tet). We found that TCF-1 is expressed in NKT cells from thymus, liver (**Fig. 1A**), spleen and lymph nodes (**data not shown**). To determine whether TCF-1 expression is regulated during NKT cell development, we also stained with CD44 and NK1.1 to be able to differentiate stage 1 (CD44^lo^ NK1.1^−^), stage 2 (CD44^hi^ NK1.1^−^) and stage 3 (CD44^hi^ NK1.1^+^) NKT cells [6], [17]. The data showed that TCF-1 expression was highest in immature stage 1, followed by stage 2 and lowest in stage 3 thymic NKT cells (**Fig. 1B** **, top**). The decreased expression of TCF-1 in mature stage 3 NKT cells was also noticeable in liver (**Fig. 1B** **, bottom**). These data document changes in TCF-1 expression in NKT cells in a developmentally sensitive manner and suggest a functional role for TCF-1 during NKT cell development.

Recently, NKT cells were redefined on the basis of expression of transcription factors PLZF and T-bet [18]. NKT2 cells express higher PLZF compared to NKT1 cells and do not express T-bet (**Fig. 1C**). Interestingly, whereas all NKT cells express TCF-1, NKT2 cells express higher level of TCF-1 compared to NKT1 cells. We conclude that the expression pattern of TCF-1 in all NKT cells and during NKT cell development advocates a role for this transcription factor.

### TCF-1-deficient mice have reduced proportion and number of NKT cells

**Figure 2:**
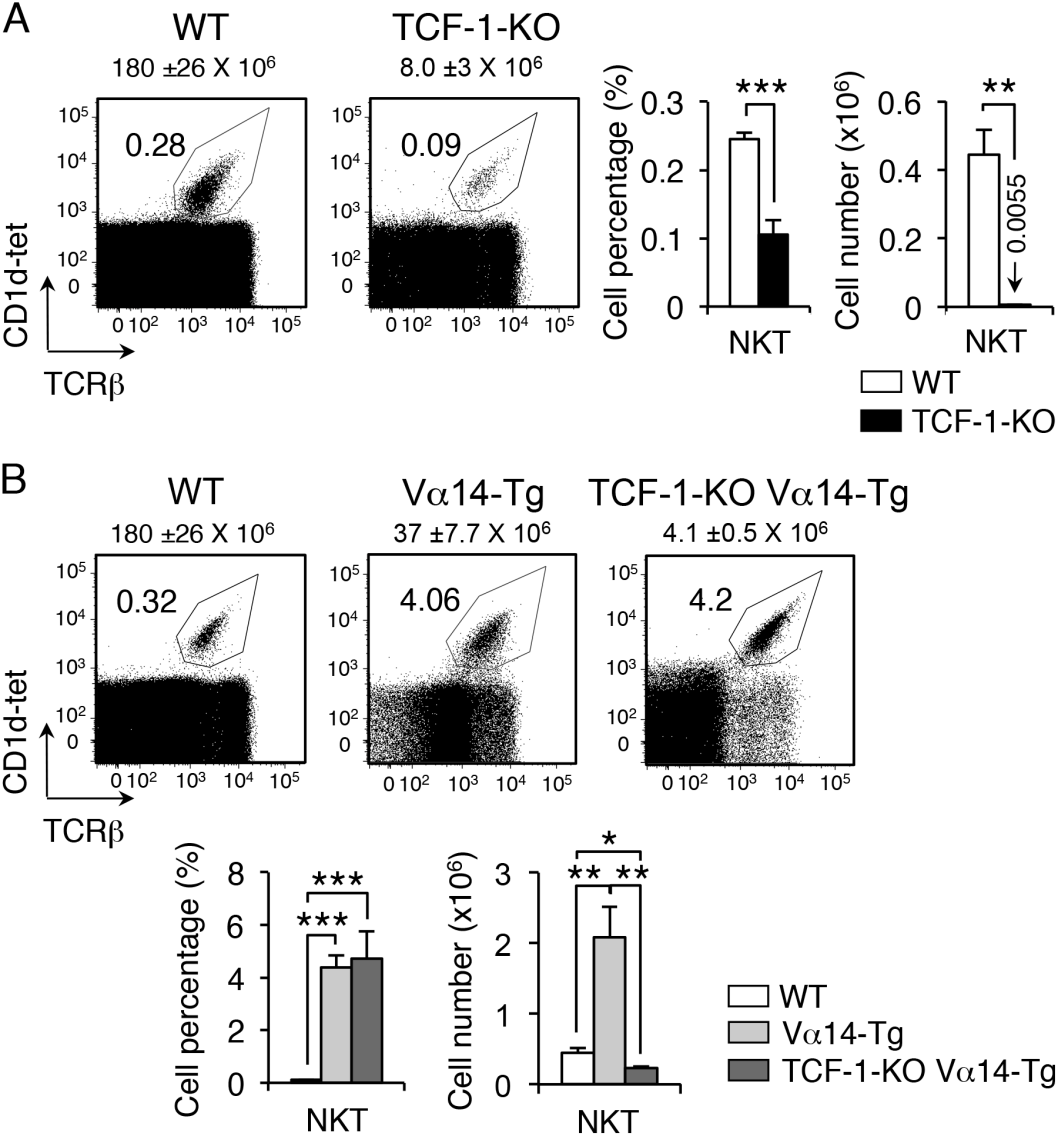
TCF-1-deficient mice have reduced proportion and number of NKT cells. Flow cytometry of thymocytes showing percent of gated CD1d-tetramer+ TCRβ+ NKT cells from wild-type (WT) and TCF-1 knock-out (KO) mice (**A**) and from WT, Vα14-Tg and TCF-1-KO Vα14-Tg mice (**B**). Representative dot plots and graphs with cell percentages and cell numbers from 4–6 mice per group are shown (mean and s.e.m.). Numbers over dot plots refer to total thymocyte cell numbers; *P<.05; **, *P*<.01; ***, *P*<.001.

To determine whether TCF-1 has a functional role in NKT cells, we analyzed thymocytes from TCF-1-KO mice. Surface staining with CD1d-tet and TCRβ showed a significant decrease of NKT cell percentage and numbers in TCF-1-deficient mice compared to control mice (**Fig. 2A**). We conclude that transcription factor TCF-1 is required for the generation of all NKT cells.

### Enforced expression of the Vα14-Tg rescues NKT cells in TCF1-deficient thymus

One reason for the reduction in NKT cells could be failure to rearrange Vα14-Jα18 TCRα chain to express the invariant TCR. To directly address this, we generated TCF-1-KO Vα14-Tg mice by breeding TCF-1-KO mice with mice expressing a rearranged Vα14-Jα18 TCRα transgene. Analysis of double-mutant (TCF-1-KO Vα14-Tg) mice showed that enforced expression of Vα14-Tg resulted in a 15-to-20-fold increase in the frequency of NKT cells compared to control mice (**Fig. 2B**). However, expression of Vα14-Tg did not rescue the absolute number of thymocytes or total number of NKT cells found in wild type mice. Together, these results suggest that TCF-1-deficiency leads to decreased generation of NKT cells due to impaired rearrangement of Vα14-Jα18 segments. As rearrangement of Vα14-Jα18 segments is temporally a distal event, we hypothesized that a decrease in the lifetime of TCF-1-deficient DP thymocytes might prevent the rearrangement of distal 3′ elements.

### Ectopic expression of Bcl-x_L_ in developing TCF-1-deficient thymocytes rescues Vα14-Jα18 rearrangements and NKT cells

**Figure 3:**
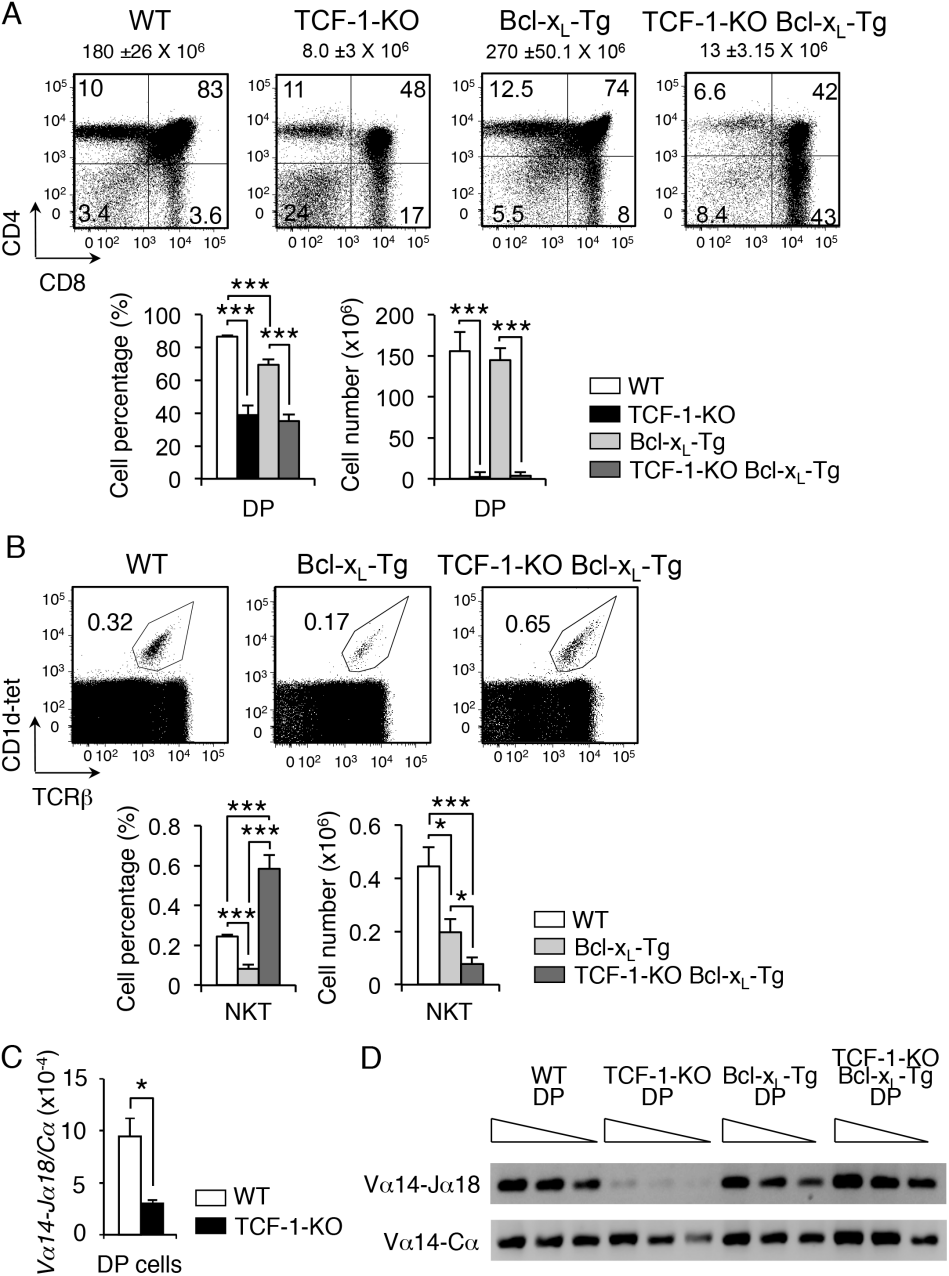
Ectopic expression of Bcl-x_L_ in developing TCF-1-deficient thymocytes rescues Vα14-Jα18 rearrangements and NKT cells. (**A**) Flow cytometry of thymocytes from WT, TCF-1-KO, Bcl-x_L_-Tg and TCF-1-KO Bcl-x_L_-Tg mice showing CD4^−^CD8^−^ (double-negative, DN), CD4^+^CD8^+^ (double positive, DP), CD4 single-positive (SP) and CD8SP thymocytes. **Top**, dot plots are representative of at least 4 different experiments. **Bottom**, graphs with DP cell percentages and cell numbers from 4 mice per group are shown (mean and s.e.m.). Numbers over dot plots refer to total thymocyte cell numbers. (**B**) Flow cytometry of thymocytes showing percent of gated CD1d-tetramer+ TCRβ+ NKT cells from WT, Bcl-x_L_-Tg and TCF-1-KO Bcl-x_L_-Tg mice. **Top**, dot plots are representative of at least 4 different experiments. **Bottom**, graphs with NKT cell percentages and cell numbers from 4 mice per group are shown (mean and s.e.m.). (**C**) Relative expression of Vα14-Jα18 rearrangements from WT and TCF-1-KO DP cells (n = 3). (**D**) Semiquantitative PCR of cDNA (1∶1, 1∶2, 1∶4 dilutions) from DP cells of WT, TCF-1-KO, Bcl-x_L_-Tg and TCF-1-KO Bcl-x_L_-Tg mice showing Vα14-Jα18 and control Vα14-Cα rearrangements (n = 3). *P<.05; ***, *P*<.001.

TCF-1-deficient DP thymocytes have reduced survival when cultured *in vitro*, which was rescued by the expression of a proximal *Lck* promoter-driven Bcl-2 transgene [16]. This report showed that survival of DP thymocytes during culture *in vitro* was regulated by TCF-1 dependent expression of Bcl-family proteins. To determine if TCF-1 regulated the lifetime of DP cells *in vivo* that led to a reduction in NKT cells, we generated TCF-1-KOxBcl-x_L_ transgenic mice (TCF-1-KO Bcl-x_L_-Tg). Representative data show that thymocyte numbers remained low in TCF-1-KO Bcl-x_L_-Tg mice (**Fig. 3A** **)**. However, analysis of NKT cell populations in TCF-1-KO Bcl-x_L_-Tg mice demonstrated a rescue of the frequency of NKT cells (**Fig. 3B**). However, the number of NKT cells remained lower than observed in control mice. We conclude that expression of Bcl-x_L_ from the proximal *Lck* promoter rescued the lifetime of TCF-1-deficient DP thymocytes *in vivo* and promoted development of NKT cells.

To further understand the role of TCF-1 in NKT cell generation, we tested the frequency of the Vα14-Jα18 rearrangement in DP cells from TCF-1-KO, TCF-1-KO Bcl-x_L_-Tg or control mice. We noted that TCF-1-deficient DP thymocytes showed significant decreased representation of Vα14-Jα18 rearrangements compared to control cells (**Fig. 3C**). The frequency of Vα14-Jα18 rearrangements was rescued in TCF-1-KO Bcl-x_L_-Tg mice (**Fig. 3D**). These data demonstrate that TCF-1-deficient DP thymocytes do not rearrange distal TCRα chains and thus do not generate a complete TCR repertoire. Incidentally, transgenic overexpression of β-catenin did not enhance the frequency of Vα14-Jα18 rearrangements (data not shown) indicating that β-catenin expression was not limiting in the definition of the lifetime of DP thymocytes. We conclude that TCF-1 is an essential component of the transcription factor profile required for proper T cell development and generation of NKT cells and T cell repertoire.

## Discussion

In this report we demonstrate that TCF-1 controls the lifetime of DP thymocytes *in vivo*, temporally distal Vα14-Jα18 rearrangements and NKT cell development. We deduce this from the observation that TCF-1-deficiency results in failure to rearrange distal Vα14-Jα18 rearrangements that impacts NKT cell generation *in vivo*. We show that, in TCF-1-deficient mice, these cells are significantly reduced in frequency and enforced expression of the NKT cell specific Vα14-Jα18 TCR transgene rescues the development of NKT cells in TCF-1-deficient mice. Finally, we demonstrate that ectopic expression of Bcl-x_L_ transgene from the proximal *Lck* promoter in TCF-1-deficient DP thymocytes extends lifetime *in vivo* to rescue Vα14-Jα18 rearrangements and NKT cells.

The transcriptional program that regulates thymic cellularity remains to be defined. TCF-1 was shown to regulate DP thymocyte survival and respond to dexamethasone challenge in a RORγt-dependent manner [15]. However, reduced thymic cellularity TCF-1-deficient mice show was not rescued by expression of genes that confer cell survival from proximal *Lck* promoter. Held *et al.* expressed Bcl-2 transgene that rescued DP thymocyte survival *in vitro* but failed to salvage thymic cellularity *in vivo* [16]. Likewise, data presented in this report show that *Lck*-driven Bcl-x_L_ transgene fails to rescue thymic cellularity. We posit that these data reflect requirement of TCF-1-dependent gene expression during early T cell development to generate thymic cellularity.

A requirement for the rearrangement of Vα14-Jα18, a temporally distal event, makes the lifetime of DP thymocytes *in vivo* a major factor in NKT cell generation. Deletion of transcription factors that regulate DP thymocyte lifetime *in vivo* show decreased NKT cell numbers, which was rescued by enforced expression of Bcl-x_L_ [19]–[23]. For example, Bcl-x_L_ overexpression in c-Myb-deficient DP cells restored survival and Vα14-Jα18 rearrangements but not NKT cell development suggesting that Vα14-Jα18 rearrangement is not sufficient for NKT cell development. Id2-deficiency is damaging to DP survival and NKT cell numbers, and both of these defects were rescued by breeding with Bim-KO mice [24]. Other transcription factors, such as c-Myc [25], have been demonstrated to regulate proliferation but not survival of NKT cells. TCF-1 expression has been linked to decreased survival of DP thymocytes *in vitro,* which was rescued by Bcl-2 expression [16]. In this report we demonstrate that TCF-1-deficient DP thymocytes have significantly fewer Vα14-Jα18 rearrangements and NKT cells. TCF-1 and LEF-1 have been shown to have redundant roles during T cell development [10]–[13]. We expect that similar overlap might be observed in the regulation of NKT cell generation in the thymus. However, in this report we show that transgenic expression of Bcl-x_L_ in DP thymocytes rescues distal TCRα rearrangement but not NKT cell numbers. It is important to note that thymic cellularity was not rescued by transgenic expression of Bcl-x_L_ suggesting that DP thymocyte lifetime is not an essential component of thymic cellularity during normal T cell development. We propose that TCF-1 controls specific gene expression required for the extended lifetime of DP thymocytes, in turn required to rearrange the distal Vα14-Jα18 segments and thereby facilitates NKT cell development.

## Methods

### Mice

TCF-1-deficient mice [26] were provided by H. Clevers (Hubrect Institute, Utrecht). Bcl-x_L_-Tg and Vα14-Tg mice were purchased from The Jackson Laboratory (Bar Harbor, ME, USA). TCF-1-KO Bcl-x_L_-Tg and TCF-1-KO Vα14-Tg were generated by crossing Bcl-x_L_-Tg and Vα14-Tg mice with TCF-1-deficient mice. All the mice used are on a C57BL/6 genetic background. Age-matched (7–10 weeks old) littermate controls or C57BL/6 mice were used in all experiments. All mice were bred and maintained in the animal facility at the National Institute on Aging (NIA). The studies were carried out in accordance with the recommendations in the Guide for the Care and Use of Laboratory Animals (NRC 2010). Mice were euthanized with carbon dioxide and tissues harvested for analysis. The protocol was approved by the Animal Care and Use Committee of the NIA Intramural Research Program, NIH. This program is fully accredited by the Association for Assessment and Accreditation of Laboratory Animal Care International (AAALAC) (File 000401), registered by the United States Department of Agriculture (51-F-0016) and maintains an assurance with the Public Health Service (A4149-01).

### Cell Preparation

Single-cell suspensions were prepared from thymuses, lymph nodes and spleens as per standard protocols. Hepatic lymphocytes were isolated from livers that were homogenized, filtered through nylon mesh and washed in PBS with 1% FBS. Cells were then resuspended in 44% Percoll (GE Healthcare Bio-Sciences AB, Uppsala, Sweden), underlaid with 66% Percoll, and centrifuged for 20 min at 2000 rpm. Cells at the interface were collected, washed, and counted.

### Flow cytometry

Cells were harvested, stained, acquired on a FACSCantoII (Becton Dickinson) and analyzed with FlowJo (Treestar). Dead cells were excluded using the Fixable Viability Dye eFluor506 (eBioscience). Antibodies conjugated to FITC, PE, PerCP Cy5.5, PE Cy7, APC, APC-Cy7 or Pacific Blue were purchased from BD Biosciences, eBioscience, BioLengend and R&D Systems. PE- or APC- conjugated mouse CD1d tetramers loaded with glycolipid PBS-57 (CD1d-tet) were obtained from the tetramer facility of the US National Institutes of Health. In brief, cells were incubated with FC block and stained with antibodies, and then fixed with 2% paraformaldehyde. For PLZF, T-bet, and TCF-1 intracellular staining, cells were permeabilized and stained with anti-PLZF (D-9) (Santa Cruz Biotechnology, Inc.) plus FITC anti-mouse (BD Biosciences), anti-T-bet PerCP-Cy5.5 (eBio4B10), both purchased from eBioscience, and with anti-TCF-1 (C63D9) (Cell Signaling) followed by goat anti-rabbit-Alexa647 (Invitrogen), using the Foxp3 Staining Buffer kit (eBioscience).

### Vα14-Jα18 rearrangement analysis

RNA from DP cells was isolated using the Qiagen RNeasy Kit (Qiagen Inc.) following the manufacturer’s instructions. cDNA was generated by RT-PCR with a SuperScript III kit (Invitrogen) and, serially diluted 1∶2, it was amplified with a forward primer specific for Vα14 (5′-GTCCTCAGTCCCTGGTTGTC-3′) paired with a downstream primer specific for the segment Jα18 (5′-CAAAATGCAGCCTCCCTAAG-3′). PCR products were separated by agarose gel electrophoresis and visualized by SYBR Safe DNA gel staining (Invitrogen). Results were normalized to Vα14-Cα expression (downstream Cα primer 5′- CAGTCAACGTGGCATCACA-3′).

### Statistics

Statistical significance was determined by the Student’s *t*-test.
